# *Spherical*: an iterative workflow for assembling metagenomic datasets

**DOI:** 10.1186/s12859-018-2028-2

**Published:** 2018-01-24

**Authors:** Thomas C. A. Hitch, Christopher J. Creevey

**Affiliations:** 1 Institute of Biological, Environmental and Rural Sciences (IBERS), Aberystwyth University, Aberystwyth, Wales SY23 3FG UK; 2 Functional Microbiome Research Group, Institute of Medical Microbiology, Uniklinik RWTH Aachen, Aachen, Germany

**Keywords:** Assembly, Metagenome, Genomics

## Abstract

**Background:**

The consensus emerging from the study of microbiomes is that they are far more complex than previously thought, requiring better assemblies and increasingly deeper sequencing. However, current metagenomic assembly techniques regularly fail to incorporate all, or even the majority in some cases, of the sequence information generated for many microbiomes, negating this effort. This can especially bias the information gathered and the perceived importance of the minor taxa in a microbiome.

**Results:**

We propose a simple but effective approach, implemented in Python, to address this problem. Based on an iterative methodology, our workflow (called *Spherical*) carries out successive rounds of assemblies with the sequencing reads not yet utilised. This approach also allows the user to reduce the resources required for very large datasets, by assembling random subsets of the whole in a “divide and conquer” manner.

**Conclusions:**

We demonstrate the accuracy of Spherical using simulated data based on completely sequenced genomes and the effectiveness of the workflow at retrieving lost information for taxa in three published metagenomics studies of varying sizes. Our results show that Spherical increased the amount of reads utilized in the assembly by up to 109% compared to the base assembly. The additional contigs assembled by the Spherical workflow resulted in a significant (*P* < 0.05) changes in the predicted taxonomic profile of all datasets analysed. *Spherical* is implemented in Python 2.7 and freely available for use under the MIT license. Source code and documentation is hosted publically at: https://github.com/thh32/Spherical.

**Electronic supplementary material:**

The online version of this article (10.1186/s12859-018-2028-2) contains supplementary material, which is available to authorized users.

## Background

Over the last 10 years, researchers have utilised high-throughput sequencing to investigate the structure and function of microbial communities from diverse environments across the globe [1–3]. While these studies have provided unique and novel insights into the workings of these microbiomes, there is a growing consensus that the tools available are not describing the full functional or taxonomic diversity that the data represents [4]. There is a need for assemblers and workflows to be developed to capture “lost” information and allow the generation of assemblies that represent the entire metagenome sampled.

Mathematically, de novo assembly of a genome falls within the class of problems for which no efficient algorithm is known (NP-hard) [5], leading to the proposal of a variety of heuristic solutions [6–9]. These have ranged from simple overlap layout consensus approaches (where sequencing reads with overlapping regions are joined together into contigs [5] to more complex approaches such as de Bruijn graphs [10]. Generally, these assemblers have been designed assuming a single genome within the data. However, as sequencing approaches for sampling the genomic information of entire microbial communities (metagenomics) began to emerge [11], it was clear that new approaches may be necessary [12].

By far the biggest issue with metagenomic sequencing datasets is the resulting uneven coverage of the taxa from the microbiome arising from the complexity and uneven distribution of species in natural microbial communities [4]. This leads to over-sequencing of dominant species in the community and heavily fragmented assemblies of the genomes of minority species, if they can be assembled at all [13].

Promising solutions to dealing with this problem utilise a ‘divide and conquer’ approach to partition the data into more easily manageable pieces [13]. For example, the data from environmental samples can be split into “bins” representing different taxa from the community [14]. Sequence reads can be sorted into bins based on properties such as kmer-frequency or the percentage of Guanine and Cytosines (GC) they contain [15]. This has the potential to increase the assembly rate of low abundance species, however it depends heavily on accurate partitioning of the data [16]. Indeed, bins of metagenomic data produced in this manner may represent a single species or an entire phylum depending on the complexity of the community [16]. SLICEMBLER [8] also implements a ‘divide and conquer’ approach by generating pre-determined “slices” of the metagenome based on coverage. Each slice is then assembled separately and frequently occurring strings are identified and used to merge contigs. This works well for deeply sequenced genomic datasets where coverage is known, however in metagenomics datasets from uncharacterised microbial communities, coverage is generally an unknown variable [17]. Previous attempts at utilising iterative binning for assembly have been developed but are not publically available [18].

Digital normalization is another commonly utilized approach for tackling complex datasets. This method, implemented in the Khmer package [19], uses kmers to identify and reduce the occurrence of highly duplicated portions of the data. This has the effect of reducing coverage of over-represented taxa, and normalising the coverage to make it more even [19]. However, while this pre-processing step allows for reduction of the datasets size, it does not reduce the complexity of the data [19] leaving subsequent assemblies prone to the problem of under-representing sections of the community.

We propose a simple but effective heuristic to address this problem that can be used in conjunction with any of the above-mentioned assembly approaches. Based on an iterative approach, our workflow (called *Spherical*) identifies the reads not accounted for in an assembly, and uses them as input for successive rounds of assemblies. *Spherical* also provides the option to minimize the resources required by assembling random subsets of the whole in a “divide and conquer” manner. The inherent downside to this approach is that by running multiple iterations of assembly, the time requirements are also increased.

## Methods

### Implementation

#### *Spherical* workflow

**Fig. 1:**
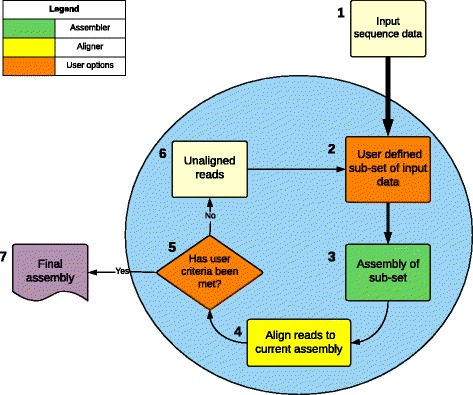
A flow-chart of the steps used by *Spherical*. The blue circle encompasses all processes that are carried out in an iterative manner until the results meet the ‘user defined criteria’. The ‘user defined criteria’ is defined as any user option which indicates a point at which *Spherical* should stop iterating. The arrows width indicates the possible decrease in file size depending on user sub-sample selection. 1; User input data (usually quality controlled sequencing files). 2; *Spherical* takes a random subset of the input sequencing data. The size of the subset is determined by user. 3; An assembly of the subset is generated. 4; The number of reads aligning to the combined assembly are determined. 5; If the number of reads aligning meets the user criteria *Spherical* will move to step 7, otherwise *Spherical* will continue to step 6. 6; Reads that do not align to the combined assembly are used as input for the next round. 7; Spherical exits and combines the individual iterations assemblies into a single file

*Spherical* uses an iterative workflow that identifies the raw data not represented in an initial assembly. This is then used as input for a subsequent assembly. This process is repeated until a pre-defined cut-off is reached, or until all the data is incorporated. The workflow is summarised in Fig. 1.

The *Spherical* workflow is composed of 5 steps:

- Step 1: Sub-sample selection

The first step in *Spherical* is the optional initial sub-sampling of the sequencing data (Fig. 1.2). This can be advantageous when working with very large datasets as it reduces the complexity of the assembly, reducing memory and time requirements. In this process a random sub-sample (defined by the option ‘-R’) is taken from the input sequencing data. Using a sub-sample size of ‘1’ selects the entire input dataset instead. If only one value is given to ‘-R’, then *Spherical* will apply this sub-sample fraction at every iteration, however the user also has the option of providing multiple different values to be used at each iteration.

- Step 2: Assembly

The sub-sample is then assembled using the assembler of choice (Fig. 1.3). By default, *Spherical* uses either Velvet [6] or ABySS [20] for assembly, it is easy to extend *Spherical* to utilise other assemblers also.

- Step 3: Alignment

When the assembly is completed, *Spherical* uses Bowtie 2 [21] to align all reads previously unaligned (in iteration 1, this is all the reads) to the contigs resulting from the assembly. All reads that do not align to the assembly produced at this iteration are saved for subsequent rounds. If a read aligns to the assembly at this point, it is considered utilised and hence excluded from the subsequent iterations. Specific commands can be passed to Bowtie2 using the ‘-u’ command followed by the commands, for example -u “-N 3 –k 2” would pass the commands to Bowtie2 to set the maximum number of mismatches in the seed to 3 and to look for at most 2 distinct valid alignments for each read.

- Step 4: Assessment

The user can define two parameters to be used by *Spherical* to determine the completeness of the assembly. The first is based on the number of iterations completed (‘-iter’). When *Spherical* has completed all iterations defined by this value, it will halt and move to Step 5. The second option is based on the proportion of reads utilised by the total assembly (‘-align 0–100’). This is calculated as the number of reads currently unaligned, divided by the total number of reads initially provided, multiplied by 100. When *Spherical* determines that the predetermined alignment rate has been reached, it will halt carrying out iterations and move to Step 5. The default options are 5 iterations or an alignment rate of 70%. If neither criteria has been met, *Spherical* will pass all unaligned reads to step 1 for another iteration of assembly.

- Step 5: Final output

Once any of the user-defined criteria for halting have been met, *Spherical* will combine the assembly from each iteration into a single file. Assembly statistics such as N50, lengths of longest and shortest contigs, the standard deviation of the lengths and the alignment rate are calculated for each iteration and for the combined assembly file. Using the ‘-m’ option merges the contig files produced by each iteration into a single file for ease of use. A final assembly which attempts to combine all the contigs created, can be carried out by specifying the ‘-f’ option.

To illustrate the outputs of the *Spherical* workflow, we carried out assemblies of both simulated and real metagenomics datasets. These are presented in the following sections.

### Simulated metagenome

The simulated metagenome examined came from a previous study [22]. This consisted of 400 species (1 genome per species) with abundances varying from 0.66% to 0.22%. This dataset was originally produced to study the effect of sequencing technologies on the assembly of metagenomic datasets. The *Spherical* assembly of this dataset used Velvet with a kmer of 31 and sub-sample size of 1 (the entire dataset).

### Real metagenomic datasets

We also present the results from the application of *Spherical* to three published metagenomic datasets: chicken caecum [23]; human oral cavity [24] and groundwater from the Yucatan peninsula (Table 1). All three metagenomic datasets were obtained from MG-RAST [25]. The Chicken cecum dataset was selected due to having a low sequencing depth and therefore allowing comparison of assembly methods on a small dataset [23]. The human oral cavity dataset was selected due to being a complex microbiome and providing moderate sequencing depth [24]. The Yucatan groundwater dataset was selected due to containing a complex microbiome and providing a high level of sequencing depth.

**Table 1:** Information on each metagenomics dataset tested using *Spherical*

Environment	Dataset size (Gbp)	MGRAST project ID
Chicken Caecum [22]	0.06	101
Human oral cavity [23]	0.63	128
Yucatan groundwater	29.00	5969

For each metagenomic dataset the table states the source environment, the datasets size in Giga base pairs (Gbp) and its MGRAST project ID

### Methods of assembly

To ensure consistency, Velvet was used to assemble each of the above datasets. Initially, an optimal kmer size was determined for each datasets by carrying out multiple initial assemblies using the entire dataset with kmer sizes of 21,31,41 and 51. Alignments of the raw reads to each of these initial assemblies was then carried out using Bowtie2 (with options: -N 1) and the number of reads aligning determined. For each dataset, the kmer size that resulted in the largest fraction of reads aligning was used as the optimal kmer for all further assemblies of that dataset (Additional file 1: Table S1).

Following determination of the optimal kmer size, each of the datasets were assembled using three approaches: 1) An assembly using only velvet; (basic assembly); 2) Initial digital normalization of the reads using Khmer, followed by an assembly with Velvet (Normalized assembly); and 3) Assembly with the *Spherical* workflow, using Velvet as the assembler (*Spherical* Assembly). These were conducted with the entire dataset (sub-set = 1).

The settings used with Velvet were the same for all three approaches using the kmer sizes determined earlier and the ‘-exp_cov auto’ option to allow Velvet to calculate the expected coverage. Meta-Velvet is a metagenomic assembler, which acts as a final step in standard Velvet assembly and so was initially investigated using the ‘meta-velvetg’ command on the output from Velvet. The options for running *Spherical* on each dataset are shown in Additional file 1 Table S5. Each assembly ran for 5 iterations apart from the chicken caecum dataset for which an optimal assembly was achieved following iteration 2.

Digital normalisation was included as a comparative approach due to its ability to reduce the size of a dataset by removal of reads consisting of redundant kmers, while keeping the datasets original complexity by retaining reads including unique kmers [19]. Digital normalization was applied using the “normalize-by-median.py” script of Khmer, with a kmer of 20, 4 hash tables of size 32e^9^ and an ideal median of 20, as suggested by the Khmer manual.

The amount of RAM necessary to carry out an assembly is a limiting factor for many large metagenomics assemblies, requiring workarounds, such as kmer normalization. For this reason the amount of RAM utilised during each assembly was monitored (Table 2).

**Table 2:** Assembly statistics comparing dataset assemblies for each method

Dataset	Method	RAM usage (Gb)	Alignment (%)	False bases (%)	Longest contig	Number of contigs
Cecum	Normalised	119	29.5	0.01	831	103,618
	Base assembly	1	29.5	0.01	831	103,618
	Metavelvet	2	29.1	0.07	831	103,618
	Spherical (1)	2	30.9	0.04	831	138,995
Oral	Normalised	14	8.1	0.01	3337	1,825,177
	Base assembly	25	13.0	0.02	4548	1,178,611
	Metavelvet	15	13.0	0.07	4548	1,178,611
	Spherical (1)	5	24.6	0.19	2380	1,053,802
Ground water	Normalised	361	52.8	3.86	117,274	5,721,819
	Base assembly	376	52.0	3.84	117,274	5,772,465
	Metavelvet	376	52.0	4.04	117,274	5,772,461
	Spherical (1)	377	59.7	2.89	117,274	13,312,643
	Spherical (0.25)	129	51.5	3.50	104,353	7,851,021
	Spherical (0.033)	107	49.8	3.78	53,836	7,145,998

The first column indicates the dataset utilized whilst the second column identified the assembly methodology. To identify the different subsampling amounts during each Spherical assembly the subsample size is stated in brackets in the method column. The final 5 columns provide information on the computational needs for each assembly (RAM usage) as well as statistics about the produced assemblies e.g. number of contigs and alignment (%)

### Assembly quality

We used a simulated metagenomic dataset generated from 400 genomes [22] to assess the quality of the de novo assemblies. Contig scores [22] were calculated by identifying the highest scoring (bitscore) match to each contig in the 400 genomes using BLASTN [26]. The formula: $\frac{coverage percentage}{100}\times percentage identity$ was used to calculate the contig score ranging from 0 to 100. A contig score of 100 indicates the entire contig precisely matches a region from the original genome and 0 means there was no matching region [22].

Contig scores cannot be calculated for real metagenomic assemblies due to requirement for a having an assembled genomes for comparison. Instead, for these datasets standard assembly statistics were calculated to provide insight in the quality of each assembly; alignment rate (defined as the percentage of sequenced reads which align to the assembly), N50 (defined as the contig length where 50% of the entire assembly is contained in contigs of equal or longer length the N50 value) and “false base rate” (defined as the percentage of bases in the assembly to which no read aligns). To calculate the “false base rate” the raw data from each dataset was aligned to each of its subsequent assemblies using Bowtie2 [21] with the option “-n 1”, which allows for a single mismatch in the seed region.

### Taxonomic identification

For all assemblies the best taxonomic matches for each contig was identified from the Bacterial and Archaeal subsets of the UNIPROT database (downloaded 10/2014) using RAPsearch (with settings: bitscore > = 40) [27, 28]. All hits found in the database were converted into a general feature format (GFF) file using the “blast2gff” command in MGKIT (http://mgkit.readthedocs.io/en/latest/) and subsequently filtered for the top non-overlapping hits with the “filter-gff” command (settings; −s 100) from MGKIT. This has the effect of resulting in a single best taxonomic match for each contig. The HTSeq-count (settings; −m intersection-nonempty) command from the HTSeq package [29] was used to identify the number of reads assigned to phyla within each assembly.

### Sub-sampling data with *Spherical*

*Spherical* allows a user to specify a subset of data to be used for each round of assembly. This is useful for the analysis of very large metagenomic datasets when RAM usage or length of time of the analysis can become issues. To demonstrate this we carried out 3 different assemblies with different sub-sampling sizes on the largest metagenomic dataset analysed (from the Yucatan Peninsula). 1) Using all the reads (sub-sample size = 1), 2) A random subsample of a quarter of all the reads (sub-sample size = 0.25) and 3) A random subsample of one thirtieth of the reads (set-sample size = 0.033). Each assembly was allowed to run for five iterations (where the same subsample size was utilised in each iteration) with the following settings: –k 41 –align 99 –iter 5. These assemblies were compared using their alignment rate, RAM usage, N50, false base rate and longest contig size.

### Effect of multiple iterations of assembly

The biodiversity within each iteration of assembly was identified and assessed for the caecum, oral and Yucatan groundwater datasets. To test if each iteration from a single dataset returned similar taxonomic profiles, a Chi-square test for homogeneity was conducted. A Z-test was then conducted between iterations which exhibited significant changes in their taxonomic profiles in order to identify which specific taxonomic groups altered in abundance. The *p*-values were then corrected using the Benjamani-Hochberg method.

## Results

### Quality analysis of resulting assemblies

We used a simulated metagenomic dataset [22] created from 400 species of varying abundance to investigate the accuracy of contigs produced by the *Spherical* workflow. For each assembled contig, within each iteration, a ‘contig score’ [22] was calculated which represents the accuracy of the reconstruction. We found that over 90% of the contigs reconstructed by *Spherical* had a contig score > = 95, representing that they were 95% identical to the original genome from which the reads were produced. Furthermore, fewer than 1% of the contigs had a score less than 50 (see Additional file 1: Table S3). The secondary iterations carried out also allowed alignment of an additional 5.25% of the raw reads compared to the base assembly. The proportion (%) of reads assigned to each genome within the dataset, base assembly and Spherical assembly are provided in Additional file 1: Table S4.

### Capture of additional information

The smallest of the microbiome datasets analysed (0.06Gbp from the Chicken Caecum) resulted in 29.5%, 29.5%, 30.9% alignment rate for the base, normalized and *Spherical* assembly respectively (Table 2). While alignment rates were very similar for the three assembly approaches used *Spherical* utilised 1.4% more of the raw reads than the other approaches but at the cost of slightly lowering the N50 (from 109 to 104) and increasing the false base rate (from 0.01% to 0.04%).

The medium-sized human oral cavity dataset (0.63 Gbp), resulted in greater variability in how the different assembly approaches performed. *Spherical* increased the alignment rate (from 13% to 24.6%) and N50 (from 190 to 234) compared to the next best approach (base assembly) (Table 2). However, the false base rate also increased slightly (from 0.02% to 0.19%) indicating the *Spherical* assembly was including a small number of bases without evidence supporting their inclusion from the raw reads.

Finally, for the largest datasets analysed (29 Gbp from Yucatan groundwater), *Spherical* resulted in an increased alignment rate (from 52.8% to 59.7%) and a decreased false base rate (from 3.86% to 2.89%). However, the N50 was reduced (from 330 to 211) in comparison to the normalised assembly. Within these datasets Meta-velvet continually produces assemblies with an increased number of false bases and was unable to outperform the base assembly, therefore meta-velvet was not investigated further.

### Sub-sampling the sequencing data

The effect of altering the sub-sample size was tested on the largest of the metagenomics datasets (Yucatan groundwater). As shown in Additional file 1: Table S5, the change in sub-sample size caused a small reduction in the quality of the resulting assembly; decreasing the false base rate from 3.86% to 3.78%, reducing the N50 from 330 to 189 and slightly reducing the alignment rate from 52.8% to 49.8% when compared to the base assembly. However, the taxonomic profile estimated from the resulting assemblies remained unchanged (Additional file 1: Figure S1). The effect of random sampling of reads for each iteration was investigated by assembling the Oral dataset 5 times with the same options (5 iterations, kmer = 31, subset size = 5). The alignment rate of both the final assemblies and individual iterations showed no variation (±0.0%) across the 5 runs.

### Multiple iterations of assembly

**Fig. 2:**
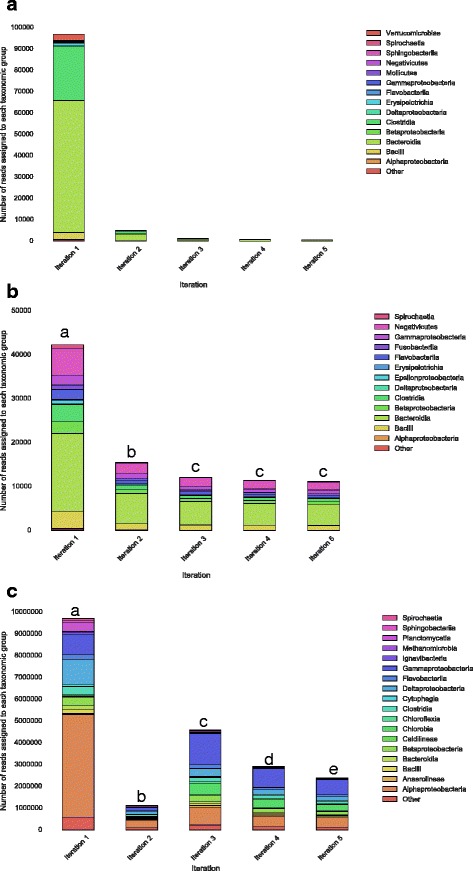
Taxonomic breakdown of each iteration for the chicken ceacum (**a**), human oral cavity (**b**) and Yucatan groundwater *Spherical* (sub-sample size = 1) (**c**) assemblies at the class level. Each bar represents the number of reads that could be assigned to a taxonomic Class within the assembly from each iteration. The colours represent different taxonomic Classes identified in the legend on the right. The letters represent the results of the significance tests where bars with the same letter are not significantly different according to the Chi^2^ test for homogeneity

The *s*econdary iterations in *Spherical* utilise previously unaligned sequencing information to assemble new genomic regions, creating more contigs for analysis (Fig. 2). The *Spherical* assemblies also had an increased number of reads aligning to annotated genes (11%, 109% and 43% in the chicken caecum, human oral and Yucatan groundwater dataset respectively (Additional file 1: Table S6)).

Whilst the taxonomic profile for the chicken caecum was consistent between iterations (Fig. 2a), we observed a significant change in between iterations both in the human oral (Fig. 2b) and Yucatan groundwater datasets (*P* < 0.05, Chi2 test for homogeneity) (Fig. 2c). The taxonomic profiles of each iteration of assembly for the Yucatan groundwater dataset was unique (Fig. 2c), suggesting novel genomic regions were being included within each iteration, increasing the accuracy of the final taxonomic composition. A two-proportion Z-test (Benjamini-Hochberg corrected) was then used to identify which taxonomic groups showed a change in abundance in the secondary iterations compared to the base assembly. The inclusion of the additional genomic regions *Spherical* assembled in the secondary iterations caused a significant change in the taxonomic profile of each dataset compared to both the base assembly and Digital normalisation (Additional file 1: Figure S1).

## Discussion

We investigated the quality of the assemblies from *Spherical* using a simulated metagenome and confirmed accurate assembly of genomic regions without a decrease in quality (Additional file 1: Table S3). Furthermore with real metagenomic datasets the secondary iterations resulted in up to 109% more reads aligning to annotated genes compared to the base assembly (Additional file 1: Table S6). This resulted in significant changes in the overall taxonomic profile predicted for both the human oral cavity (*P* < 0.05) and Yucatan groundwater metagenomes (*P* < 0.05). Unsurprisingly, the contigs from the subsequent iterations of assembly were more fragmented, resulting in a lower N50, however they represented information that would otherwise have been missed if a single round of de novo assembly had been used.

The sub-sampling option of *Spherical* results in a reduction of up to 70% in the RAM required to assemble the metagenomes with only a small reduction in alignment rate compared to the base assembly (Table 2). This will be attractive to research groups who lack access to sufficient computational facilities to carry out assemblies of complex metagenomes in single step. The inherent downside to this approach is that by running multiple iterations of assembly, the time requirements are also increased.

## Conclusion

*Spherical* is a workflow that allows the capture and use of data that might otherwise be missed in a metagenomics dataset assembly. It allows the construction of high quality assemblies of metagenomics datasets without restricting users to particular tools or assembly approaches. Implemented in Python it is simple to use and freely available to the scientific community under an MIT license. If applied to novel or previously published datasets it provides the opportunity to reveal novel biological information that may otherwise have been missed.

## Additional file

Additional file 1: Table S1. Effect of kmer size on assembly. For each dataset (column 1) the alignment rate (%) (column 3) of raw data aligning back to assembly produced using different kmers (column 2) was assessed. **Table S2**. Effect of kmer size across iterations of assembly using the simulated dataset. The percentage of raw data aligning to each iterations assembly was identified and iterations stopped once the alignment rate was under 0.1%. **Table S3**. Analysis of the quality of contigs produced in each iteration of assembling the simulated dataset using contig scores. The contig score identifies the percentage accuracy of a contig compared to the genomes used to create the simulated metagenome. We present the percentage of contigs from each iterations assembly with contig scores gereater than 95 or less than 50. **Table S4**. Percentage of reads assigned to each of the 400 genomes within the simulated dataset, base assembly and Spherical assembly of the simulated dataset. **Table S5**. Assembly statistics comparing dataset assemblies for each method. The first column indicates the dataset utilized whilst the second column identified the assembly methodology. Due to Spherical having a sub-sampling option the size of the sample utilized by Spherical was stated for each assembly in column 5. The final 6 columns provide information on the computational needs for each assembly (RAM usage) as well as statistics about the produced assemblies e.g. number of contigs and alignment (%). **Table S6**. The number of reads aligning to genes within the Spherical iterations assembling each dataset. **Figure S1**. The taxonomic variations at the phylum level between each experimental assembly method for each dataset. Each bar represents the number of reads that could be assigned to a taxonomic Phyla within each assembly method for the datasets. The legend identifies which Phyla is represented by each colour. (ZIP 562 kb)
