# Supplementary material for: Spherical: an iterative workflow for assembling metagenomic datasets
Source: BMC Bioinformatics. 2018 Jan 24;19:20. doi: 10.1186/s12859-018-2028-2 (PMC5781261; doi:10.1186/s12859-018-2028-2)
Supplement: Additional file 1: Table S1. — Effect of kmer size on assembly. For each dataset (column 1) the alignment rate (%) (column 3) of raw data aligning back to assembly produced using different kmers (column 2) was assessed. Table S2. Effect of kmer size across iterations of assembly using the simulated dataset. The percentage of raw data aligning to each iterations assembly was identified and iterations stopped once the alignment rate was under 0.1%. Table S3. Analysis of the quality of contigs produced in each iteration of assembling the simulated dataset using contig scores. The contig score identifies the percentage accuracy of a contig compared to the genomes used to create the simulated metagenome. We present the percentage of contigs from each iterations assembly with contig scores gereater than 95 or less than 50. Table S4. Percentage of reads assigned to each of the 400 genomes within the simulated dataset, base assembly and Spherical assembly of the simulated dataset. Table S5. Assembly statistics comparing dataset assemblies for each method. The first column indicates the dataset utilized whilst the second column identified the assembly methodology. Due to Spherical having a sub-sampling option the size of the sample utilized by Spherical was stated for each assembly in column 5. The final 6 columns provide information on the computational needs for each assembly (RAM usage) as well as statistics about the produced assemblies e.g. number of contigs and alignment (%). Table S6. The number of reads aligning to genes within the Spherical iterations assembling each dataset. Figure S1. The taxonomic variations at the phylum level between each experimental assembly method for each dataset. Each bar represents the number of reads that could be assigned to a taxonomic Phyla within each assembly method for the datasets. The legend identifies which Phyla is represented by each colour. (ZIP 562 kb) [file 12859_2018_2028_MOESM1_ESM.zip › Supplementary tables and figures.pdf]

Supplementary tables and figures:

Supplementary Table 1: Effect of changing the kmer size provided to the assembler. For each dataset (column 1) the alignment rate (%) (column 3) of raw data aligning back to assembly produced using different kmers (column 2) was assessed.

Dataset	Kmer size	Alignment rate (%)
Chicken caecum	21	29.05
	31	26.71
Human Oral cavity	21	8.11
	31	12.97
	51	19.51
Yucatan groundwater	31	41.75
	51	13.59

Supplementary Table 2: Effect of changing the kmer size across iterations of assembly using the simulated dataset. The percentage of raw data aligning to each iterations assembly was identified and iterations stopped once the alignment rate was under 0.1%.

Method	Statistic	Iteration 1	Iteration 2	Iteration 3	Iteration 4	Iteration 5
Effect of decreasing kmer size	Kmer	61	51	41	31	NA
	Alignment rate	0.82%	0.61%	0.16%	0.01%	NA
Effect of Increasing Kmer size	Kmer	31	51	61	NA	NA
	Alignment rate	35.12%	0.43%	0.03%	NA	NA
Effect of consistant kmer size	Kmer	31	31	31	31	31
	Alignment rate	35.12%	0.95%	0.55%	0.12%	0.1%

Supplementary Table 3: Analysis of the quality of contigs produced in each iteration of assembling the simulated dataset using contig scores. The contig score identifies the percentage accuracy of a contig compared to the genomes used to create the simulated metagenome. We present the percentage of contigs from each iterations assembly with contig scores gereater than 95 or less than 50.

Contig score	Iteration1	Iteration 2	Iteration 3	Iteration 4	Iteration 5
**Greater than 95**	99%	94%	94%	97%	97%
**Less than 50**	<1%	<1%	<1%	<1%	<1%

Supplementary Table 4: Percentage of reads assigned to each of the 400 genomes within the simulated dataset, base assembly of the simulated dataset and Spherical assembly of the simulated dataset.

Supplementary Table 4 provided as additional Excel file.

Supplementary Table 5: Assembly statistics comparing dataset assemblies for each method. The first column indicates the dataset utilized whilst the second column identified the assembly methodology. Due to *Spherical* having a sub-sampling option the size of the sample utilized by *Spherical* was stated for each assembly in column 5. The final 6 columns provide information on the computational needs for each assembly (RAM usage) as well as statistics about the produced assemblies e.g. number of contigs and alignment (%).

Supplementary Table 5 provided as additional Excel file.

Supplementary Table 6: The number of reads aligning to genes within the *Spherical* iterations assembling each dataset.

Dataset	Iteration 1	Iteration 2	Iteration 3	Iteration 4	Iteration 5
**Chicken Caecum**	$1.64x10^5$	$1.1x10^4$	$3.4x10^3$	$2.3x10^3$	$1.4x10^3$
**Human Oral Cavity**	$1.3x10^5$	$4.4x10^4$	$3.3x10^4$	$3.1x10^4$	$3.0x10^4$
**Yucatan groundwater**	$1.3x10^8$	$2.7x10^7$	$1.2x10^7$	$8.6x10^6$	$7.8x10^6$

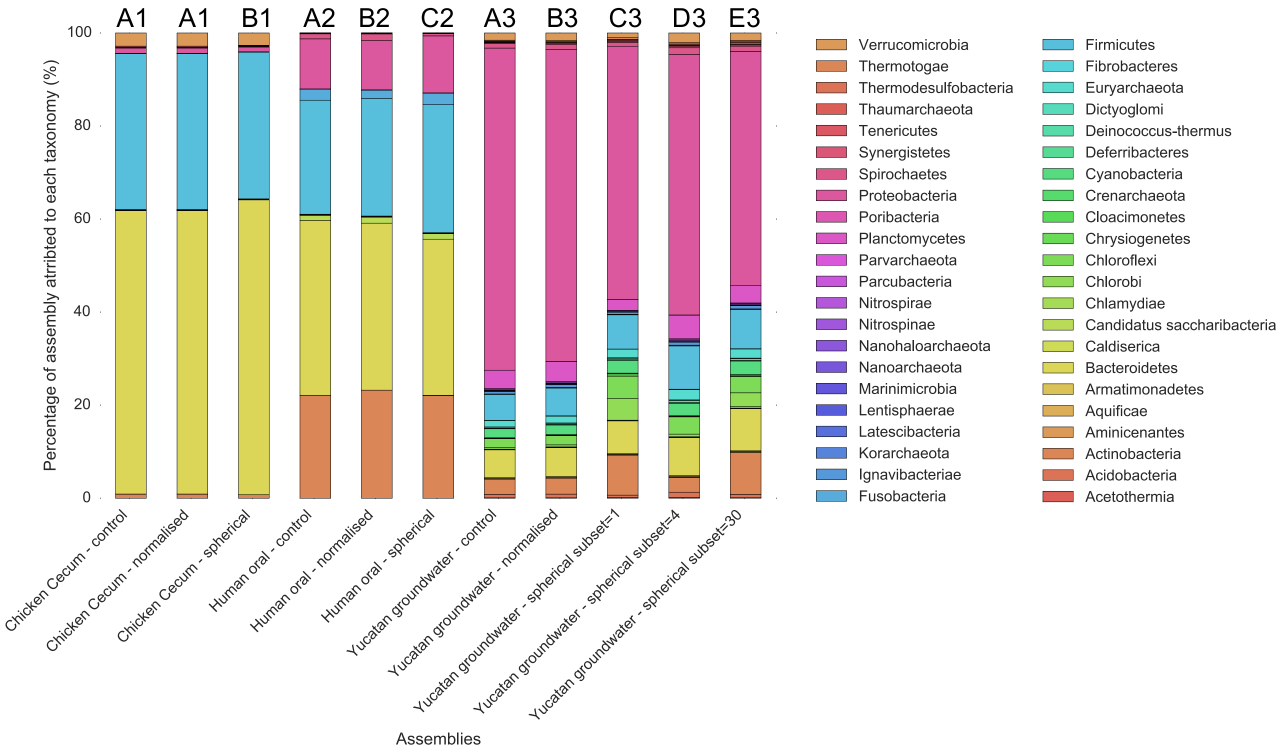

Supplementary Figure 1: The taxonomic variations at the phylum level between each experimental assembly method for each dataset. Each bar represents the number of reads that could be assigned to a taxonomic Phyla within each assembly method for the datasets. The legend identifies which Phyla is represented by each colour.

Supplementary Table 7: Results from two-proportion Z-test, after correction with the Benjamini-Hochberg method, on the Human Oral dataset to identify which taxonomic groups were driving the shift in taxonomic profile between the base assembly and each additional iterations assembly. Significant (adjusted P<0.05) results are bold. Each letter (A,B,C) indicates a taxonomic profile assigned to one or more iterations via the homology tests, Figure 4.

Taxonomic group	A V B	Fold change	A V C	Fold change
**Betaproteobacteria**	**0.02565**	-1.47	**0.02238**	-1.56
**Bacteroidia**	**0.00008**	-1.37	**0.00002**	-1.49
**Gammaproteobacteria**	**0.02088**	-1.30	**0.01681**	-1.39
**Spirochaetia**	0.09382	-1.63	0.11951	-1.99
**Clostridia**	**0.02565**	-1.56	**0.02884**	-1.82
**Epislonproteobacteria**	0.08062	-1.53	0.08062	-1.71
**Bacilli**	**0.01681**	-1.40	**0.01310**	-1.48
**Other**	0.10792	-1.63	0.13661	-2.00
**Eryipelotrichia**	0.13542	-1.60	0.16035	-2.03
**Fusobacteria**	**0.03174**	-1.30	**0.02565**	-1.37
**Negativicutes**	**0.00677**	-1.35	**0.00413**	-1.45
**Flavobacteria**	**0.02565**	-1.41	**0.02238**	-1.50
**Alphaproteobacteria**	0.14104	-1.54	0.16560	-2.00
**Deltaproteobacteria**	0.16109	-1.63	0.15747	-1.73

Supplementary Table 8: Results from two-proportion Z-test, after correction with the Benjamini-Hochberg method, on the Yucatan groundwater dataset to identify which taxonomic groups were driving the shift in taxonomic profile between the base assembly and each additional iterations assembly. Significant (adjusted $P<0.05$) results are bold. Each letter (A,B,C,D,E) indicates a taxonomic profile assigned to one or more iterations via the homology tests, Figure 4.

Taxonomic group	A V B	Fold change	A V C	Fold change	A V D	Fold change	A V E	Fold change
**Chlorobia**	**0.00071**	-1.90	**8.2e-13**	+2.31	**7.3e-13**	+2.12	**1.1e-12**	+1.93
**Betaproteobacteria**	**0.00001**	-1.99	**2.8e-06**	-1.13	**5.7e-07**	-1.34	**3.1e-07**	-1.40
**Bacteroidia**	**0.00008**	-2.04	**0.00063**	-1.37	**0.00006**	-1.55	**0.00004**	-1.64
**Gammaproteobacteria**	**1.7e-07**	-1.87	**3.02e-12**	+1.15	**8.2e-13**	-1.03	**8.2e-13**	-1.11
**Flavobacteria**	**0.00022**	-2.34	**0.00001**	-1.10	**2.8e-06**	-1.33	**2.5e-06**	-1.42
**Spirochaetia**	**0.00018**	-1.96	**0.00294**	-1.44	**0.00040**	-1.63	**0.00023**	-1.71
**Sphingobacteria**	**0.00009**	-1.80	**0.00301**	-1.42	**0.00079**	-1.70	**0.00044**	-1.77
**Bacilli**	**0.00001**	-1.69	**0.00013**	-1.23	**0.00001**	-1.39	**0.00001**	-1.50
**Ignavibacteria**	**0.00079**	-2.06	**0.00741**	-1.47	**0.00305**	-1.82	**0.00218**	-1.93
**Methanomicrobia**	**0.00009**	-1.65	**0.00094**	-1.23	**0.00022**	-1.42	**0.00020**	-1.53
**Caldilineae**	**0.00003**	-1.30	**0.00002**	+1.20	**0.00001**	+1.03	**0.00001**	-1.08
**Cytophagia**	**0.00002**	-1.63	**0.00199**	-1.40	**0.00040**	-1.65	**0.00022**	-1.71
**Chloroflexia**	**0.00003**	-1.48	**0.00003**	+1.06	**0.00001**	-1.10	**0.00002**	-1.24
**Clostridia**	**5.7e-07**	-1.64	**0.00001**	-1.21	**1.4e-06**	-1.37	**1.4e-06**	-1.50
**Planctomycetia**	**2.6e-06**	-1.87	**0.02705**	-1.97	**0.00236**	-2.28	**0.00082**	-2.35

**Other**	**2.8e-07**	-1.74	**0.00005**	-1.36	**3.2e-06**	-1.58	**2.03e-06**	-1.67
**Alphaproteobacteria**	**0.00022**	-2.38	**2.8e-06**	-1.80	**5.7e-07**	-2.12	**0.00005**	-2.19
**Anaerolineae**	**3.8e-06**	-1.47	**1.8e-06**	+1.24	**2.6e-06**	+1.08	**3.2e-07**	-1.01
**Deltaproteobacteria**	**0.00003**	-2.02	**3.3e-05**	-1.45	**1.8e-06**	-1.69	**8.6e-07**	-1.79
